# An Implantable Ultrasonically-Powered Micro-Light-Source (µLight) for Photodynamic Therapy

**DOI:** 10.1038/s41598-019-38554-2

**Published:** 2019-02-04

**Authors:** Albert Kim, Jiawei Zhou, Shayak Samaddar, Seung Hyun Song, Bennet D. Elzey, David H. Thompson, Babak Ziaie

**Affiliations:** 1 Department of Electrical and Computer Engineering, Temple University, Philadelphia, PA USA; 2 School of Electrical and Computer Engineering, Purdue University, West Lafayette, IN USA; 3 Birck Nanotechnology Center, West Lafayette, IN USA; 4 Department of Chemistry, Purdue University, West Lafayette, IN USA; 5 Department of Electronic Engineering, Sookmyung Women’s University, Seoul, Republic of Korea; 6 Department of Comparative Pathobiology, Purdue University, West Lafayette, IN USA

## Abstract

Photodynamic therapy (PDT) is a promising cancer treatment modality that can selectively target unresectable tumors through optical activation of cytotoxic agents, thus reducing many side effects associated with systemic administration of chemotherapeutic drugs. However, limited light penetration into most biological tissues have so far prevented its widespread adoption beyond dermatology and a few other oncological applications in which a fiber optic can be threaded to the desired locations via an endoscopic approach (e.g., bladder). In this paper, we introduce an ultrasonically powered implantable microlight source, μLight, which enables *in-situ* localized light delivery to deep-seated solid tumors. Ultrasonic powering allows for small receiver form factor (mm-scale) and power transfer deep into the tissue (several centimeters). The implants consist of piezoelectric transducers measuring 2 × 2 × 2 mm^3^ and 2 × 4 × 2 mm^3^ with surface-mounted miniature red and blue LEDs. When energized with 185 mW/cm^2^ of transmitted acoustic power at 720 kHz, μLight can generate 0.048 to 6.5 mW/cm^2^ of optical power (depending on size of the piezoelectric element and light wavelength spectrum). This allows powering multiple receivers to a distance of 10 cm at therapeutic light output levels (a delivery of 20–40 J/cm^2^ light radiation dose in 1–2 hours). *In vitro* tests show that HeLa cells irradiated with μLights undergo a 70% decrease in average cell viability as compared to the control group. *In vivo* tests in mice implanted with 4T1-induced tumors (breast cancer) show light delivery capability at therapeutic dose levels. Overall, results indicate implanting multiple µLights and operating them for 1–2 hours can achieve cytotoxicity levels comparable to the clinically reported cases using external light sources.

## Introduction

**Figure 1:**
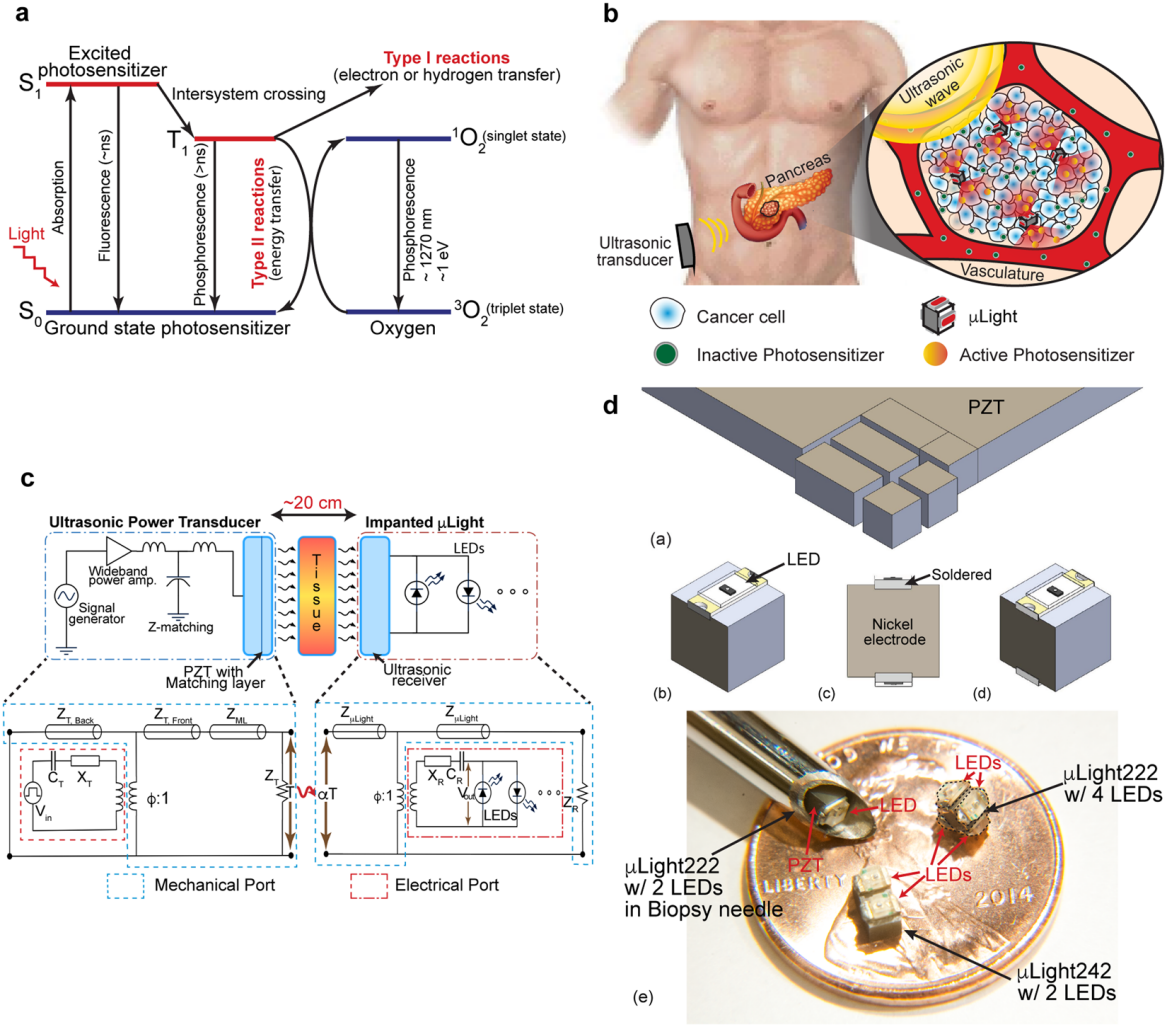
(**a**) Working mechanism of PDT: Light exposure excites the photosensitizer molecule from ground singlet state (S_0_) to an excited state (S_1_). The molecule in S1 may undergo intersystem crossing to an excited triplet state (T_1_) then either form radicals via Type 1 reaction or transfers its energy to triplet oxygen (^3^O_2_) to form a singlet oxygen (^1^O_2_), which is major cytotoxic agent via Type II reaction^4,5^. (**b**) Illustration of implantable µLight in a deep-seated tumor: ultrasonic waves applied from an external transducer travel through tissue to trigger light generation by the μLight, thus activating a pre-delivered photosensitizer, which in turn generates reactive oxygen species (ROS) to kill cancer cells. (**c**) Ultrasonic powering: Schematics of the power transfer link and its theoretical electromechanical model^33^ © 1999 IEEE. Reprinted, with permission, from Sherrit, S., Leary, S. P., Dolgin, B. P. & Bar-Cohen, Y. Comparison of the Mason and KLM equivalent circuits for piezoelectric resonators in the thickness mode. 1999 IEEE Ultrason. Symp. Proceedings, Int. Symp. (**d**) Fabrication process for the μLight: (**a**) dice PZT into 2 × 2 × 2 mm^3^ and 2 × 4 × 2 mm^3^ pieces, (**b**) solder one LED between the nickel electrodes, (**c**) solder another LED with reverse polarity on the opposite side (more LEDs can be soldered on the two remaining sides), (**d**) parylene-C coat the device for passivation, (**e**) optical photographs of fabricated prototypes.

Chemotherapy along with radiation and surgery is one of the three pillars of cancer treatment^1^. In clinical practice, it consists of systemic administration of cytotoxic chemicals, which due to a lack of selectivity, simultaneously attack many normal cells in the body, causing well-known side effects such as fatigue, hair loss, pain, constipation, nausea, and blood disorders. To minimize the side effects, researchers have developed targeted cancer therapies that aim to maximize drug interaction, specifically with cancer cells, while causing minimal damage to the healthy cells (immunotherapy being an example of latest developments in this area)^2,3^. One such technique is photodynamic therapy (PDT), which relies on selective optical activation of cytotoxic drug using a light source^4,5^. This provides spatial control through a synergistic combination of two otherwise non-toxic components: a photosensitizer (PS) and light. The photosensitizer generates cytotoxic agents only under light illumination with a particular wavelength. When absorbing photons, the photosensitizer becomes activated from the ground singlet state (S_0_) to an excited singlet state (S_1_). As it relaxes back to the ground state, the molecule can undergo two kinds of reactions, Type I and Type II^6,7^. Type I is the reaction with a molecule, transferring a hydrogen atom to form radicals that interact with oxygen to produce singlet oxygenated products (^1^O_2_). Type II refers to direct energy release to oxygen to form singlet oxygen (^1^O_2_) (Fig. 1a). Both reactions create reactive oxygen species (ROS, with a half-life of <40 ns and interaction radius of <20 nm) in a highly localized manner to attack the malignant cells^8^.

Despite its remarkable advantages as compared to standard chemotherapy, limited light penetration depth (<5 mm) at the wavelengths required by typical photosensitizers (500–600 nm) have restricted the applications of PDT to superficial dermatologic legions^9^. A possible approach to circumvent this limitation is deployment of wireless miniature light sources within the tumor^10,11^. Bansal. *et al*. recently presented radio-frequency powered miniature light sources, which could reach up to 5 cm^12^. Similarly, near-field communication LED chip in sandwiched by bio-adhesive polydopamine-modified poly(dimethylsiloxane) (PDMS) has introduced as metronomic PDT modality^13^. A few other relative easy to access organs (e.g., bladder, lung, esophagus, head and neck, glioblastoma, prostate cancer) could treated by an interstitial fiber optics^14–17^.

In this work, we outline an alternative approach of an ultrasonically powered implantable microlight source, μLight, to overcome the light delivery depth limitation and. The device converts the incoming ultrasonic wave into *in-situ* light. The use of ultrasonic powering enables for optimal size and form factor such that the device can be inserted using a biopsy needle. In addition, ultrasonic powering allows for deep tissue powering (>10 cm), not achievable with the traditional inductive methods^18^. The μLight can be used in primary or metastatic unresectable solid tumors. An important example of the former is pancreatic cancer in which 80% of tumors are unresectable and easily recurrent. The μLight can be used to ablate or shrink the tumor such that it can be removed as seen in clinical cases by Huggett *et al*.^19^. Metastatic liver tumors (commonly originating from colorectal, lung, or ovarian cancers) are examples of the latter. RF ablation is commonly used to treat such tumors; however, these procedures are associated with considerable morbidity and due to their invasiveness, they cannot be repeated. μLight allows for dose fractionation (i.e., the treatment can be administered in several sessions), thus both reducing the side effects and increasing the efficacy. Finally, recent advances in transmitting ultrasound across the skull to treat/ablate intracranial tumors has created the opportunity to place the μLights in the tumor through a burr hole and administer PDT without the need for a large and invasive craniotomy^20^.

## Results and Discussion

Figure 1b illustrates the microlight source, μLight, implanted in a deep-seated tumor (e.g., pancreas). An external ultrasonic transducer generates an acoustic wave which travels through tissue to the implant. Once energized, the onboard LEDs turn on and activate pre-delivered photosensitizers. The μLight consists of a small ultrasonic receiver (lead zirconate titanate, PZT-5A) with dimensions of 2 × 2 × 2 mm^3^ and 2 × 4 × 2 mm^3^ and super-bright surface mount LEDs (e.g., red, blue, or combinations). Such dimensions and form factors allow for easy insertion using a biopsy needle (gauge 8). The choice of LEDs is mainly dictated by its luminous intensity and excitation wavelengths of photosensitizers^21–23^. The μLight can carry up to four LEDs on the surfaces of the 2 × 2 × 2 mm^3^ embodiments or up to six on the 2 × 4 × 2 mm^3^ one; thus, capable of activating a large volume around the device.

### Ultrasonic Powering

Although inductive powering has been a clinically accepted wireless powering scheme for many decades^24^; at mm-scale it suffers from small efficiency, short transmission range, misalignment sensitivity, and manufacturing difficulty (e.g., it’s difficult to wound micro-coils at µLight dimensions). Inductive powering offers excellent performance only when the transmitter and receiver coils have a similar form factor, are aligned in parallel, and are in close proximity (e.g., cochlear implants). Ultrasonic powering is an attractive alternative wireless powering scheme since it offers unique advantages such as misalignment insensitivity, higher efficiency at mm-scale receiver size, and a larger penetration depth (>20 cm)^18,25–30^.

Figure 1c shows the ultrasonic powering schematic for *in situ* light delivery and its theoretical Krimholtz, Leedom, Mattaei (KLM) electrical equivalent circuit model^31–33^. The transmission system has three blocks; the ultrasonic transmitter, tissue, and the implanted receiver (i.e., μLight). The ultrasound transmitter is powered by an amplified and impedance matched sinusoidal signal from a function generator. At the input electrical port of the KLM model, amplified signal (*V*_*in*_) passes through an electrode capacitance (*C*_*T*_) and frequency-dependent reactance (*X*_*T*_) before being converted to mechanical motion represented via the electromechanical coupling and modeled as a transformer (*ϕ:1*). The two faces of PZT transmitter and the acoustic matching layer are modeled as quarter wavelength transmission lines (*Z*_*TS*_, *Z*_*ML*_). The mechanical input power to the receiver is denoted by *αT* where *T* is the transmitted mechanical power and *α* is the tissue attenuation factor (*α* = *e*^−2*μx*^, *μ* is the attenuation coefficient and *x* is the implantation depth). It is important to limit the transmitted ultrasonic power intensity to values approved by the FDA for ultrasonic imaging applications to avoid cavitation and heat induced damage to normal tissue (peak intensity of 720 mW/cm^2^)^34^. Similar to the transmitter, the receiver is also modeled as quarter-wave transmission lines (*Z*_*μLight*_*s*) and mechanical-electrical conversion similarly by a transformer (*ϕ:1*). The output electrical voltage source (*V*_*out*_) is again modelled with a parallel plate electrode capacitance (*C*_*R*_) and a frequency dependent acoustic reactance (*X*_*R*_). Note that the series reactance, *X*_*R*_ becomes zero at resonant frequency operation; thus, the electrical part of receiver model could be modeled as an AC source with a source capacitance, *C*_*R*_.

To maximize the power transfer efficiency, the transmission link is operated at resonant frequency of the piezoelectric elements determined by the thickness of PZT (*λ/2* = *t*, 1.09 MHz for 2 mm-thick PZT-5A). However, the simple aforementioned equation is true only if the aspect ratio (width/thickness) of transducer is greater than 10. In cuboidal form factors, such as the receivers used in the μLight, the resonant frequency deviates from the plate approximation due to Poisson’s ratio and other associated mode coupling effects of each axis^35,36^. The measured resonant frequency for the µLight with 2 × 2 × 2 mm^3^ and 2 × 4 × 2 mm^3^ receivers were 586 kHz and 650 kHz, respectively (Supplementary Fig. 1)^37^. Table 1 summarizes mechanical and electrical characteristics of the receiver components, i.e., PZT and LEDs. Figure 1d shows the fabrication process which consists of dicing a PZT wafer followed by soldering the LEDs and coating the device with a biocompatible polymeric thin film. (See the Methods sections for details).

**Table 1:** Characteristics of the components used in the μLight.

Characteristics of the PZT-5A receiver		Electrical and optical characteristics of LEDs	
Longitudinal velocity of sound in PZT-5A	4,350 m/s	Peak wavelength for the red LED	655 nm
Thickness of the µLight PZT	2 mm	Peak wavelength for the blue LED	470 nm
Resonant frequency for the PZT, μLight242	672 kHz	Turn-on voltage for the red LED	1.55 V
Resonant frequency for the PZT, μLight242	720 kHz	Turn-on voltage for the blue LED	2.8 V

**Figure 2:**
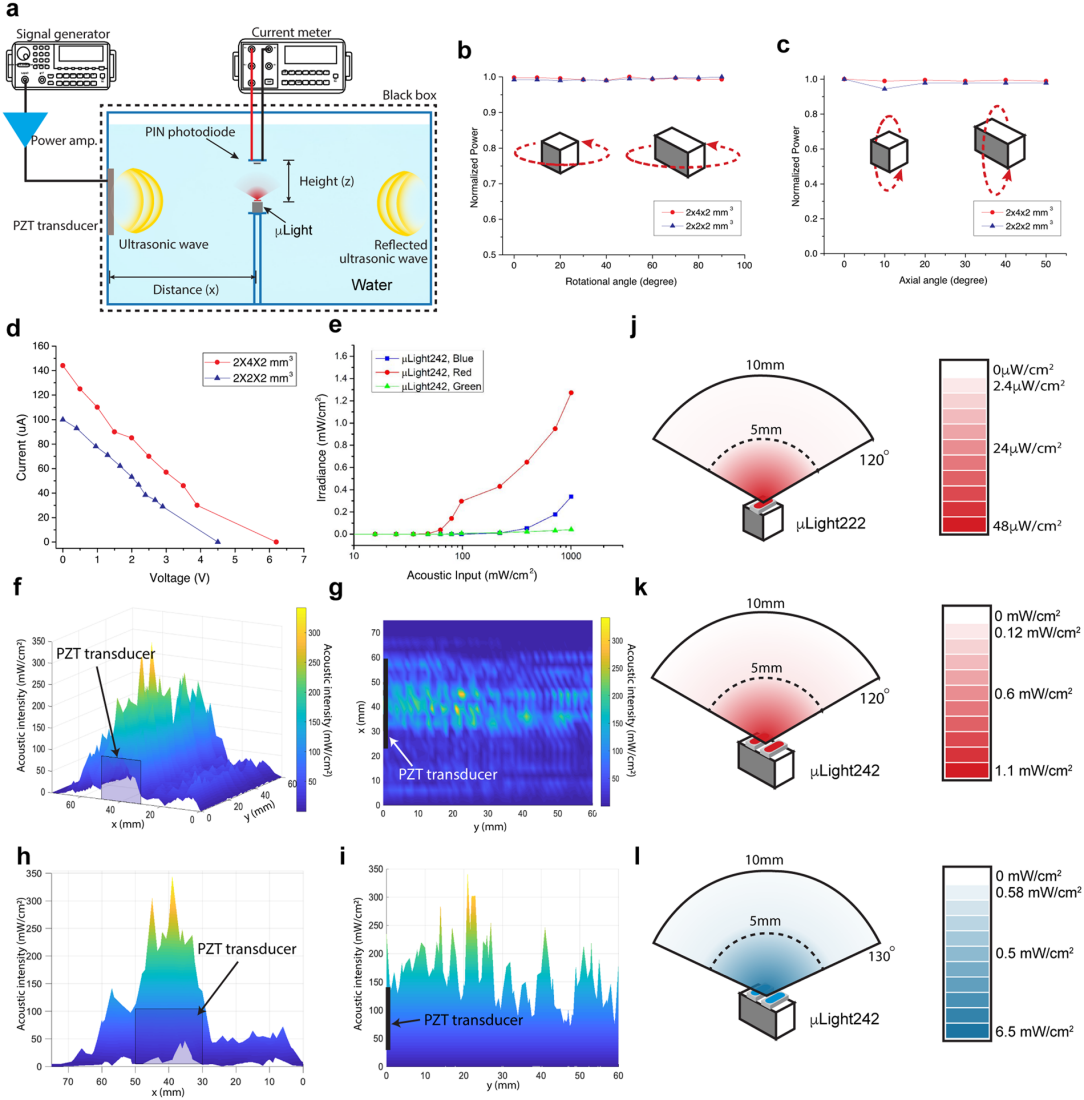
(**a**) Water tank experimental setup, (**b**) rotational angular misalignment, (**c**) axial angular misalignment, (**d**) I–V characteristics at transmitted ultrasonic intensity of 185 mW/cm^2^, (**e**) input acoustic intensity vs. µLight activation response, (**f**) non-reflected ultrasound propagation across the frontal region of the transmitter (**g**–**i**) ultrasound propagation profiles seen on XY, YZ, and XZ planes, (**j**) light irradiance of μLight222, red at various vertical/transverse distances and angles, (**k**) light irradiance of μLight242, red at various vertical/transverse distances and angles, (**l**) light irradiance of μLight242, blue at various vertical/transverse distances and angles.

The μLight was first characterized in terms of acoustic penetration depth and receiver directionality in water (a common model for soft tissue due to their similar acoustic properties (*Z*_*water*_ =1.48 MRayl, *Z*_*tissue*_ = 1.63 MRayl, mean value for human tissue^38^). For each receiver size (2 × 2 × 2 mm^3^ and 2 × 4 × 2 mm^3^), devices were submerged in a water tank (dimension of 50 × 50 × 30 cm^3^) and excited by an ultrasonic transmitter (3.6 × 3.6 cm^2^) positioned within the near-field range, Fig. 2a. A signal generator (566 kHz sinusoidal wave for the μLight222 and 650 kHz for the μLight242) with a wideband power amplifier (ENI A300, Electronics & Innovation, Ltd. NY, USA) was used to drive the transmitter. The transmitted intensity was set to 185 mW/cm^2^, below the FDA limit for imaging applications. The acoustic power was measured with an ultrasound power meter (Precision Acoustic, Inc., UK). Figure 2b,c shows the normalized received electrical output power as a function of rotational and axial angular misalignment with respect to the ultrasonic input wave. As can be seen, the received output remains constant over 90-degree rotational and 50-degree axial angular misalignments (the axial angular was measured only up to 50 degrees due to experimental setup limitations), confirming the misalignment insensitivity of the receivers, as expected from the omni-directionality of ultrasound^28^.

The received electrical power for various load resistances at a constant transmitted ultrasonic intensity of 185 mW/cm^2^ was also measured, Fig. 2d. The 2 × 2 × 2 mm^3^ and the 2 × 4 × 2 mm^3^ PZT receivers were able to generate 108 μW (2 V and 54 μA) and 171 μW (3 and 57 μA), respectively, sufficient to power the LEDs. The μLight222 device can power red LEDs (*V*_*F*_ = 1.8 V) and the μLight242 can power either red, blue, or green (*V*_*F*_ = 2.8 V) LEDs. At the 185 mW/cm^2^ transmitted ultrasonic intensity, the maximum electrical power outputs were 2.2 mW for the μLight222 receiver and 3.3 mW for the μLight242 one (efficiencies of 1.7% and 2.7%, respectively, $\eta =|{P}_{output}/{P}_{input}|$)^27^.

### Light Delivery

The light generation performance of the μLight was evaluated by measuring the converted light intensity in a water tank setup placed inside of a black box to block the ambient light (Fig. 2a and Supplementary Fig. 2). The light irradiances of the μLight222 (1 LED facing the photodiode) and μLight242 (2 LEDs facing the photodiode) were measured using a PIN photodiode (VBP104S, Vishay Ltd.). In our case, we chose three different types of LEDs (AlGaAs for a red LED, InGaN for a blue and green LED) based on the output power measurements and forward turn-on voltages (1.8 V and 2.8 V). The measured current from the photodiode (2400 sourcemeter, Keithley Instruments) was converted back to light irradiance level. Figure 2e shows light irradiances for μLight242 (red, blue, and green LEDs) under increasing transmitted acoustic intensities. The difference in threshold voltage for μLight with different LEDs was firstly investigated. Irradiated lighting intensity in a non-reflection condition was firstly measured with μLight placed at 20 mm away from the transducer. When the device was turned on, the light intensity started increasing. The acoustic intensity generated near the transducer surface was measured for comparison. As shown in the Fig. 2e, the device with red LEDs can be turned on when the input acoustic intensity was around 60 mW/cm^2^, whereas the acoustic intensity needed for device with blue LED to be turned on is around 390 mW/cm^2^. It’s important to note that this acoustic input is not the transmitted intensity (which will be measured later), rather received intensity. As a result, the μLight with red LED requires relatively lower input to turn on as compared to blue or green LED.

Following the µLight activation intensity measurement, the transmitted acoustic intensity was also evaluated. It is important characterize since the light irradiance of μLight at various location within the body depends on the transmitted acoustic intensities. The non-reflective ultrasound propagation across the frontal region of the transmitter was investigated by measuring the acoustic intensity with a fiber-optics hydrophone (Precision Acoustic, UK), Fig. 2f–i. While the transmitter dimensions and wavelength define the near-field (~15 cm for the ultrasonic transmitter size of 3.6 × 3.6 cm^2^ at 650 kHz, *N* = *D*^2^/4*λ*, where *N* is near-field distance, *D* is the transducer diameter, *λ* is the ultrasonic wavelength), the acoustic intensities within the near-field region varies and maximize along the center axis; thus, it is much preferable region for the µLight.

To evaluate the performance of μLight within the proximity of transmitter (i.e., within the near-field region) in a reflective condition, the light intensity measurement was then repeated in a 5 cm diameter semispherical water tank with transducer placed at the bottom to create reflections in the water. The maximum light intensities for μLight222 were 48 μW/cm^2^ measured at the surface of the red LED and subsequently attenuated in the water according to the inverse square law (1/*d*^*2*^). The μLight242, meanwhile, could deliver light at much higher intensities, i.e., 1.1 mW/cm^2^ for the red LED and 6.5 mW/cm^2^ for the blue LED (both measured at the surface). Since the emitted light intensities are significantly lower than conventional external light sources (up to 1 W/cm^2^ for lasers)^39^, adequate therapeutic light energy dose (e.g. 20 J/cm^2^ for Foscan)^6^ requires longer operation time (e.g., a one hour operation with μLight at 5.5 mW/cm^2^ irradiance can achieve a light dosage of 20 J/cm^2^).

The light transmission was also measured *ex vivo* using soft bovine tissue (Supplementary Fig. 3). The light intensity emitted by the LEDs was measured at different tissue thicknesses. The results show blue light attenuates by 67%, when passing through 1.3 mm of soft tissue; whereas red light attenuates 50% less (33%) across 1.3 mm thick tissue. This was expected since red light interaction with soft tissue is known to be less than that of the shorter wavelength blue light.

### *In vitro* and *In vivo* Tests

The μLight devices were tested *in vitro* as to evaluate their functionality to initiate a cytotoxic response in a cell culture environment. Our choice of photosensitizer (PS) was verteporfin which has been studied for pancreatic cancer^19^. Verteporfin has two peak absorptions around wavelength of 400–450 nm and 665–685 nm^40^. Based on the device characterizations (e.g., light delivery and operational threshold), we selected μLight242 with two red LEDs (wavelength range of 637–671 nm with a peak at 655 nm) for *in vivo* experiment.

**Figure 3:**
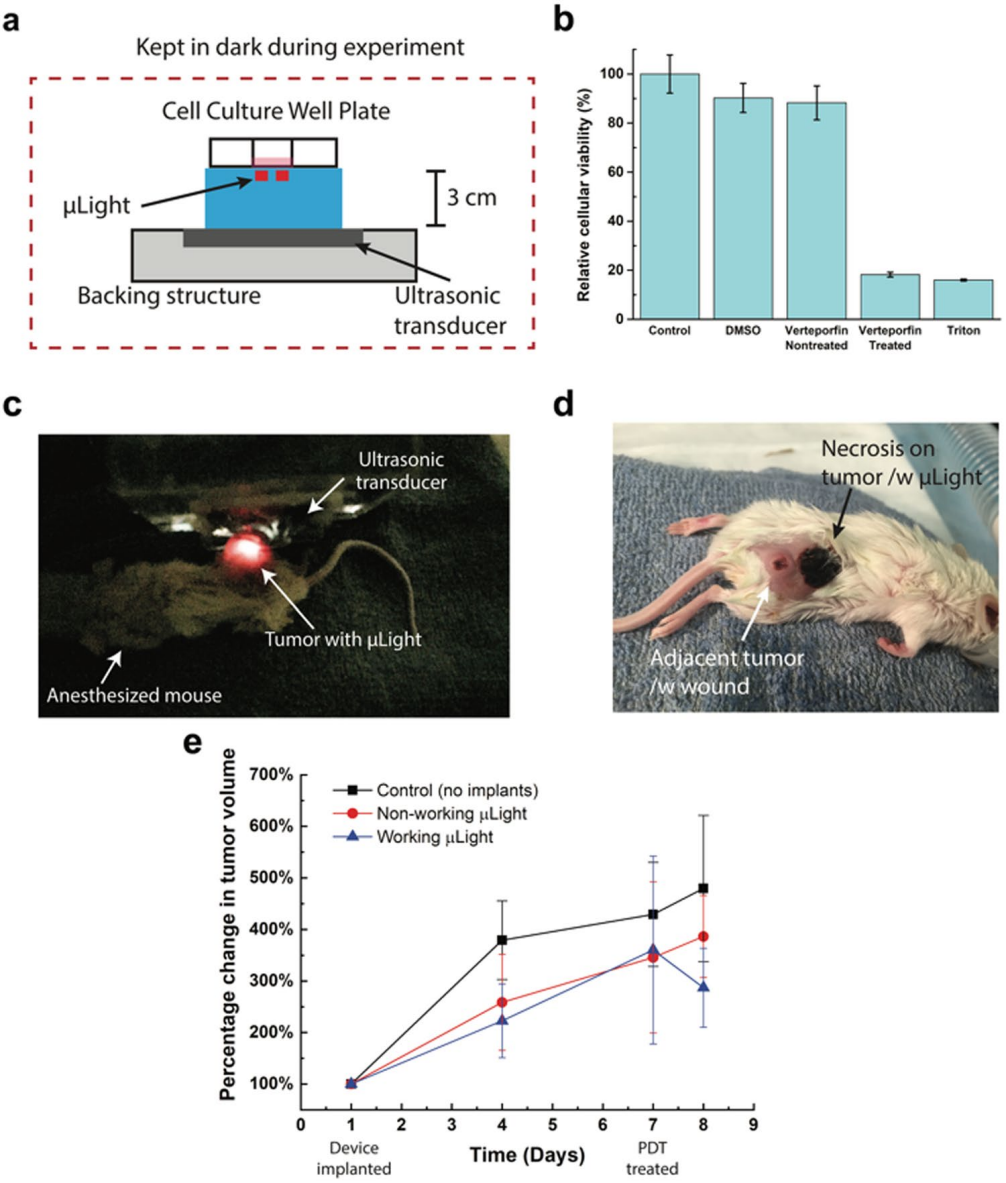
(**a**) *In vitro* experimental setup, (**b**) cytotoxicity assay of experimental groups: control (no treatment), cell culture media with ultrasonic treatment; DMSO only; verteporfin added but non-treated; expectation (verteporfin introduced cell culture media with active μLights by ultrasonic treatment); and Triton X100 treated (added to kill cells), (**c**) *in-vivo* experimental setup: μLight was implanted in mouse and excited/powered via ultrasound, (**d**) optical image of the mouse (24 hours after PDT treatment), (**e**) Tumor volume change with respect to the size when device was implanted. PDT treatment with μLights was conducted 7 days after the surgery allowing for wound healing.

Dimethyl sulfoxide (DMSO) was used as the solvent for verteporfin and proper dosage of was determined prior to the test (see Methods section for details). HeLa cells (human cervical cancer cells) were selected for *in vitro* validation since it has been widely used for proof-of-concept evaluation. Cells were seeded onto culture dishes and were allowed to grow for 24 hours before the experiment. Five different cell groups were tested: (1) cells with only cell culture media (control); (2) cells with 1 mL cell culture media and DMSO (0.14% v/v); (3) cells with cell culture media and verteporfin without photodynamic treatment (1 mL cell culture media with 4 μM verteporfin and 0.14% v/v DMSO); (4) cells with cell culture media and verteporfin with photodynamic treatment (1 mL cell culture media with 4 μM verteporfin and 0.14% v/v DMSO); and finally (5) cells are killed by Triton X100. For group 4, during ultrasound treatment, all samples were kept in the dark to eliminate the effect of the ambient light that could activate verteporfin. Figure 3a shows the experimental setup in which two red μLight242s (655 nm) were placed under each well and powered through a 3 cm-thick 0.5% w/v agarose gel to mimic the power transfer through soft tissue. The ultrasonic power at 185 mW/cm^2^ was turned on for 30 minutes and generated light from the μLight could be visually detected/observed. The light dose delivered for each well was around 4 J/cm^2^. Figure 3b shows the relative cell viability for the five experimental groups. As can be seen, 30 minutes of photodynamic therapy using μLight could induce 70% decrease in average cell viability as compared to the cells that were just exposed to verteporfin (without the device being turned on, group 3) and 82% decrease as compared to the control (group 1) which shows a significant difference (one-way ANOVA, p < 0.05). In addition, through a separate control experiment, no significant difference (ANOVA) in cytotoxicity was introduced by pre-treated Verteporfin and we confirmed that the cytotoxic effect is only due to the *in situ* light generation. (See details on Supplementary Fig. 4).

We move forward with *in vivo* animal test after the positive *in vitro* results. We demonstrated the *in vivo* functionality of μLight in BALB/c mouse model. Figure 3c shows the *in vivo* experimental setup used to validate the capability of light delivered by μLight through the soft tissue. Tumor was induced in BALB/cJ mice by implanting cultured 4T1 cells in hind flank. We chose the 4T1 model in order to test the device in an immunocompetent setting where the immune system can participate in tumor eradication. Human pancreatic cancer cells would have to be tested in immunocompromised mice. After tumor had grown to average size of 200 mm^3^ in volume among mice, a μLight242 was implanted in the interstitial space around the tumor of sixteen mice (see Methods section for details). Allowing for the wound to recover for a week, near the implantation site, adjacent tumor cells migrated forming a second tumor and completely surrounding the device, resulting a deployment of the device inside tumor tissue. Upon the complete recovery, first PDT was initiated by intra-tumoral injection of Verteporfin on day 7. Out of eighteen mice, six mice were picked to a control group (no implants), eight mice were for a group with µLight, but no ultrasonic treatment, and four mice for a treatment group. We excluded the mice which died before the experiments and mice that reduced tumor naturally. Overall, each group had average of six animals.

For treatment group, μLight was activated wirelessly using ultrasound, after 15-minute of Verteporfin injection. The treatment time was set to 30 minutes (2 J/cm^2^ light dose). This protocol was designed to consider the drug deliver in the system as well as µLight performance. The μLight was generating light while the ultrasonic transducer supplied power from the outside, Fig. 3c. Tumor size was measured with a caliper and optical pictures were taken periodically. While the tumor size in control groups (non-operating µLight and no implants) shows faster growth, the test group with working µLight shows a decrease in average tumor size (~20% shrinkage, P = 0.009^*^) on day 8, Fig. 3e. Figure 3d is the picture taken 24 hours after the PDT treatment. The results and picture show necrotic tissue resulting from successful PDT (the small wound on the migrated adjacent tumor was observed before the PDT treatment and was probably a fungating legion). Necrosis was restricted to the tumor tissue where the device was implanted (device was surrounded by necrosis and thus not visible). The affected volume was limited by the light penetration depth into the tumor parenchyma, with larger tumors requiring multiple devices to achieve complete removal. Although additional studies are needed to evaluate the parameters required for optimal efficacy, this preliminary *in vivo* result indicates that μLight can be powered while being implanted and trigger the photosensitizer.

## Discussion

The expected treatment time estimated from the previously investigated clinical trials for PDT^6,9,21^ shows that μLight can have clinical relevance. Table 2 summarizes the current commercially available photosensitizers, their tested dosage (0.1–2 mg/kg delivered orally or intravenously), and light wavelength (lower wavelengths around 408 nm show more efficacy) and energy densities (between 3–200 J/cm^2^). After a post-infusion time (30 min to 24 h, determined by drug administration), various light energy densities have been used to treat lung, early-stage head/neck, and bladder cancers among others. Most of the studies utilized higher light energy densities using the external system (e.g., floor lamp, laser, etc.). However, a few studies suggested that the higher light intensities does not increases the PDT efficacy^21^. The 1.3 J/cm^2^ could be achieved from 20 minutes from μLight242 with 2 on-board LEDs. Twenty minutes of treatment time for PDT is not significantly longer than conventional PDT. Moreover, the actual treatment time could be even shorter with the μLight, since the light intensities reported from the clinical trials were not measured inside the tumors. For tumors larger than 0.5 cm^3^ in volume, multiple μLights should be implanted to cover the entire tumor volume (in a similar manner the brachytherapy seeds are placed inside a prostate cancer tumor). A recent study^13^ has presented the feasibility of applying low power light source to metronomic photodynamic therapy (low lighting dosage modality with extended treatment time).

**Table 2:** Clinically available photosensitizers and anticipated treatment times^6,9,21^.

Platform	Substance	Wavelength (nm)	Concentration (mg/kg)	Light Energy (J/cm^2^)	Treatment time (hours)			Target organ
					µLight222 red	µLight242 red	µLight242 blue	
Porphyrin	HpD	408, 630	0.8–2.0	200–300	1157.4–1736.1	50.5–75.8	9.1–13.7	Lung, Head, Neck, Bladder
Porphyrin	ALA	410, 635	100 umol/L	3–4	34.7–138.9	1.5–6.1	0.13–13.7	Head, Neck, Bladder, Prostate
Chlorine	Temoporfin	652	0.1–0.2	20	115.7	5.1	0.9	Lip, Oral, Lung, Stomach, Pancreatic
Chlorine	Talaporfin sodium	408, 664	>2.5	100	578.7	25.3	4.3	Head, Neck
Chlorine	HPPH	408, 665	2.5–6	44.4–133.2	256.9–770.8	11.2–25.3	1.9–5.7	Lung
Padoporfin	Phthalocyanine	670–763	—	100	578.7	25.3	4.3	Lip, Pharynx, Larynx, Tongue
Porphyrin	Verteporfin	690	—	100	578.7	25.3	4.3	Eye
Texaohyrins	Lutexaphyrin	732	0.5–2	2.5–150	—	—	—	Breast

## Methods

### Fabrication of µLight

The fabrication of μLight is illustrated in Fig. 1d. The lead zirconate titanate (PSI-5A4E, Piezo Systems Inc., MA, USA) was machined/diced into pieces of desired dimensions: 2 × 2 × 2 mm^3^ (henceforth known as μLight222) and 2 × 4 × 2 mm^3^ (henceforth known as μLight242), Fig. 1d(a). Super-bright red (*λ* = 640 nm, InGaN, APT2012SRCPRV, Kingbright Ltd.) or blue (*λ* = 470 nm, AlGaAs, LTST-C191TBKT, Lite-On Technology Corp.) LEDs were subsequently soldered on top of the nickel electrode surfaces of the PZT, Fig. 1d(b). Additional LEDs were soldered on other sides (multiple LEDs can be assembled to increase the angular coverage, up to 4 LEDs on μLight222 and up to 6 LEDs on μLight242), Fig. 1d(c). The red and blue LEDs have emission angles of 120° and 130°, respectively, allowing for a total angular coverage of nearly 360° by using multiple LEDs. Finally, the device was coated with 5 μm of parylene-C for passivation and biocompatibility, Fig. 1d(d). Several fabricated devices are shown in Fig. 1d(e).

### Preparation of cell culture media

Media solution for *in vitro* experiment was prepared using Gibco DMEM (Dulbecco Modified Eagle Media) supplemented with 10% fetal bovine serum, 1% penicillin-streptomycin and 1% L-glutamine.

### Administration of verteporfin

0.5 mg verteporfin (Sigma Aldrich) was first dissolved in 250 μL DMSO (Sigma Aldrich). The solution was well stirred for complete dissolution. During device functionality evaluation, for treated group, 1.44 μL of verteporfin-DMSO solution was added to 998.56 μL of cell culture media to create a mixture of 1 mL with 4 μM concentrated verteporfin.

### Verteporfin dosage assay

The concentration range at which the drug itself is non-toxic to cells was determined by measuring lethal dose, 50% (LD50) value. HeLa cells were seeded at a density of 1.5 × 104 cells/well in a 96 well plate 24 hours before treatment. Cells were then treated with varying concentration of verteporfin (2.7 mM to 0.084 mM) (see Supplementary Fig. 5). The untreated wells had DMSO which corresponds to the amount of DMSO in the well with the highest concentration of the drug. Cells were incubated for 24 hours before analyzing the LD50 value using CellTiter 96 Aqueous One Solution Cell Proliferation Assay (MTS) according to the protocol. As the result, a concentration of 4 μM was selected which shows no cytotoxicity to the HeLa cells.

### Preparation of tissue phantom

TRIS borate- EDTA buffer solution (Fluka Analytical) was diluted by deionized water to 10% v/v. to prepare the base solution. Agarose powder (Sigma Aldrich) was dissolved at 100 °C while stirring in deionized water at a concentration of 0.5% w/v. Dissolved agarose solution was then stored in a vacuum chamber under 1 Torr for 10 min. The degassed pre-gel solution was poured into a glass beaker for cooling under the room temperature.

### Procedure for animal handling and *in vivo* experiments

Animal preparation and handling was in strict compliance with our approved Purdue University’s IACUC protocol (approval #: 1112000342 on 1/16/2016). Female BALB/cJ mice were obtained from Jackson Laboratory. Each group (control, dummy device, and test) had six to eight animals. 4T1 cell lines were obtained from American Type Culture Collection (ATCC) and cultured according to ATCC protocols. One million tumor cells were suspended in physiologic buffer and implanted into the hind flank area of mice. The device was sterilized with 70% ethanol for 30 minutes and surgically implanted when the size of the tumor was approximately 200 mm^3^.

Ultrasound excitation treatment was conducted after complete healing of the wound. To prepare the photosensitizer, 1 mg verteporfin was dissolved 0.25 mL DMSO following by 10 times dilution to 2.5 mL solution. For each mouse, 25 μL of verteporfin solution was injected resulting in a verteporfin dose of 0.5 mg/kg per mouse (weight of each mouse was around 20 g). The mouse was anesthetized using 3% isoflurane in a reclamation chamber and placed on a heating pad to maintain the body temperature during implantation as well as the µLight operation. For each experimental group, the tumor size for three mice was collected and measured.

Following the *in vivo* experiments, 21 days after tumor implantation, the mouse was euthanized per approved IACUC protocol. During the treatment, hair on the targeted site was shaved. Ultrasound gel (Medline) was applied to the skin as transmission media between mouse and ultrasound transmitter. Ultrasound transmitter was in touch with the gel during the treatment.

### Statistical analysis

For *in vitro* and *in vivo* studies, experimental data are presented as mean ± standard deviation, obtained from at least three independent measurements. For *in vitro* cell culture validation, the means comparison of cytotoxicity among groups were analyzed for statistical significance using one-way analysis of variance (ANOVA) followed by post-hoc Tukey test for comparing multiple conditions. For *in vivo* experiments, two-way ANOVA was used to compare control groups and treatment group. Difference was considered significant when *p < 0.05. Statistical analysis was performed using OriginPro software.

## Supplementary information

Supplementary Info File #1
