# Supplementary material for: An Implantable Ultrasonically-Powered Micro-Light-Source (µLight) for Photodynamic Therapy
Source: Sci Rep. 2019 Feb 4;9:1395. doi: 10.1038/s41598-019-38554-2 (PMC6362227; doi:10.1038/s41598-019-38554-2)
Supplement: Supplementary file 1 — Supplementary Info File #1 [file 41598_2019_38554_MOESM1_ESM.pdf]

An Implantable Ultrasonically-Powered Micro-Light-Source (μLight) for Photodynamic Therapy

A. Kim[1], J. Zhou[2,3], S. Samaddar[4], S. H. Song[5], Bennett D. Elzey[6], D. H. Thompson[4], and B. Ziaie[2, 3*]

[1]Department of Electrical and Computer Engineering, Temple University, Philadelphia, PA, USA
[2]School of Electrical and Computer Engineering, Purdue University, West Lafayette, IN, USA
[3]Birck Nanotechnology Center, West Lafayette, IN, USA
[4]Department of Chemistry, Purdue University, West Lafayette, IN, USA
[5]Department of Electronic Engineering, Sookmyung Women's University, Seoul, Republic of Korea
[6]Department of Comparative Pathobiology, Purdue University, West Lafayette, IN, USA

Correspondence to B. Ziaie (bziaie@purdue.edu)

Supplementary Figures

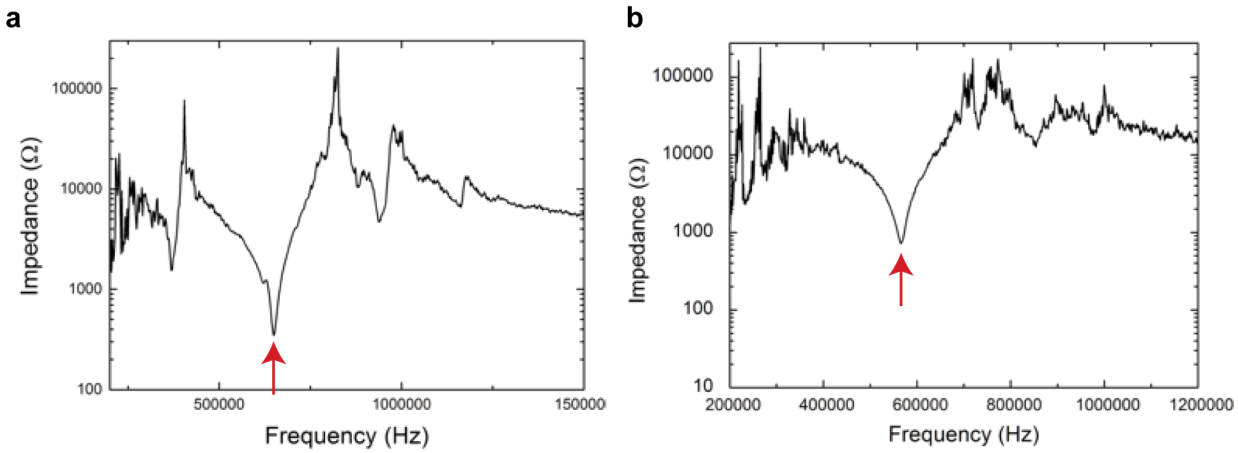

Supplementary Fig. 1 | Impedance over various frequency. Measured resonant frequency (the frequency of the lowest impedance) of (a) 2×2×2 mm^3 PZT receiver was 586kHz, and (b) 2×4×2 mm^3 receivers was 650 kHz

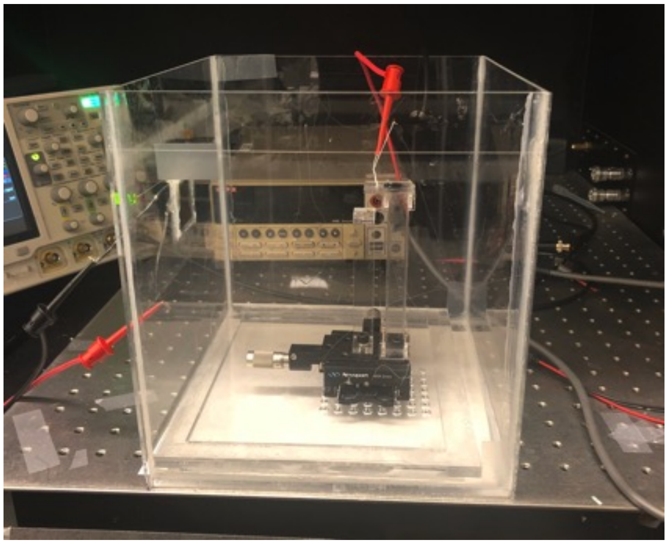

Supplementary Fig. 2 | Light delivery measurement setup

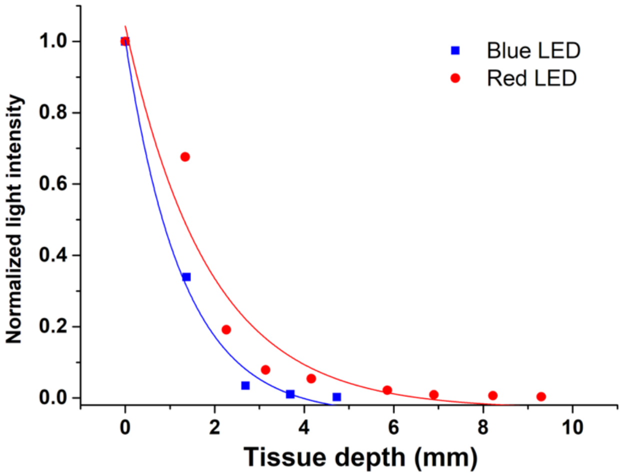

Supplementary Fig. 3 | Light penetration through bovine tissue

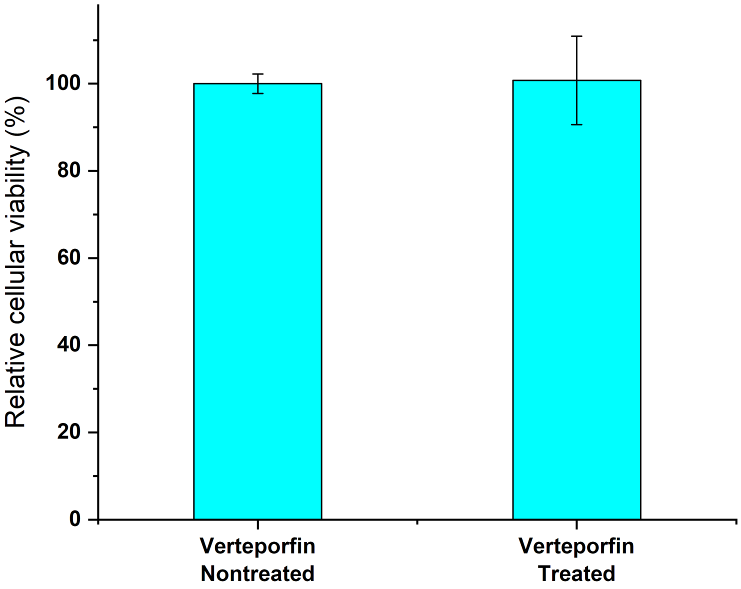

Supplementary Fig. 4 | Cytotoxicity assay of photo excited sample. Verteporfin was treated with μLight242 using the same *in vitro* experiment protocol for 30 minutes before transferred to HeLa cells. The Verteporfin concentration is 72 μg/mL and DMSO concentration was 1% wt. The relative cellular viability was examined for experimental groups.: verteporfin added but non-treated; verteporfin treated with red light prior to introduce to cell culture media. Light treated verteporfin doesn't show an increase of cytotoxicity due to short life time of ROS.

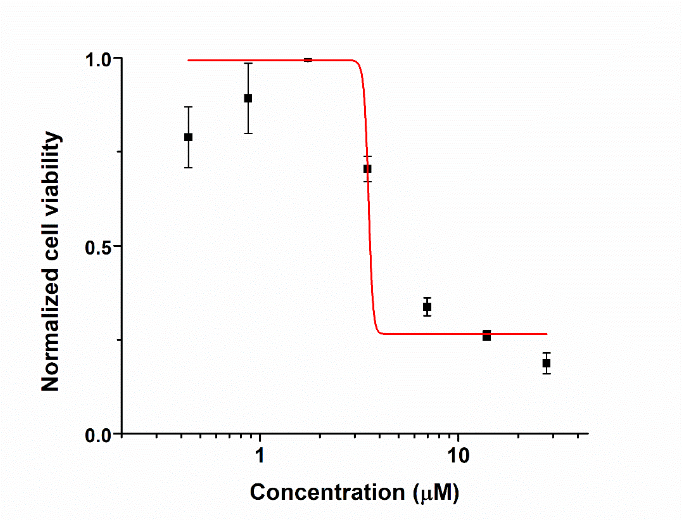

Supplementary Fig. 5 | LD50 value measurement: Relative cell viability vs. verteporfin concentration
